# Matrix metalloproteinase‐2, ‐7, and ‐9 activities in dogs with idiopathic pulmonary fibrosis compared to healthy dogs and dogs with other respiratory diseases

**DOI:** 10.1111/jvim.15970

**Published:** 2020-12-04

**Authors:** Merita Määttä, Henna P. Laurila, Saila Holopainen, Kaisa Aaltonen, Liisa Lilja‐Maula, Sanna Viitanen, Minna M. Rajamäki

**Affiliations:** ^1^ Faculty of Veterinary Medicine, Department of Equine and Small Animal Medicine University of Helsinki Helsinki Finland

**Keywords:** canine, gelatinases, lung, matrilysin

## Abstract

**Background:**

Canine idiopathic pulmonary fibrosis (CIPF) is a chronic, interstitial lung disease that mainly affects West Highland white terriers (WHWTs) and is characterized by excessive deposition of extracellular matrix (ECM) in the lung. Matrix metalloproteinases (MMPs) participate in remodeling of ECM.

**Objectives:**

To compare metalloproteinase‐2, ‐7 and ‐9 activities in blood or bronchoalveolar lavage fluid (BALF) samples or both of CIPF WHWTs with healthy WHWTs, healthy dogs of other breeds, and dogs with other lung diseases and determine if these MMPs could be used as diagnostic and prognostic markers for CIPF.

**Animals:**

Forty‐four CIPF WHWTs, 24 dogs with chronic bronchitis (CB), 17 with eosinophilic bronchopneumopathy (EBP), 10 with bacterial pneumonia, 39 healthy WHWTs, and 35 healthy dogs of other breeds.

**Methods:**

Cross‐sectional observational study. Pro‐MMP and active MMP activities were analyzed by zymography.

**Results:**

In serum, significantly higher (*P* < .01) pro‐MMP‐7 activities were observed in CIPF WHWTs compared to healthy dogs of other breeds, dogs with CB and dogs with EBP. In BALF of CIPF WHWTs, both pro‐MMP‐9 and pro‐MMP‐2 activities were significantly higher (*P* < .01) compared to healthy WHWTs, but these differences were not detected in plasma. The CIPF WHWTs had significantly higher (*P* < .05) activities of pro‐MMP‐9 compared to dogs with CB and of pro‐MMP‐2 compared to dogs with CB and EBP. No statistically significant prognostic factors were observed in CIPF WHWTs.

**Conclusions and clinical relevance:**

Serum MMP‐7 and BALF MMP‐2 and ‐9 potentially may be useful diagnostic markers but not prognostic markers for CIPF.

**Abbreviations**
ANCOVA: analysis of covariance
BALF: bronchoalveolar lavage fluid
BP: bacterial pneumonia
CB: chronic bronchitis
CI: confidence interval
CIPF: canine idiopathic pulmonary fibrosis
EBP: eosinophilic bronchopneumopathy
ECM: extracellular matrix
HR: hazard ratio
HRCT: high‐resolution computed tomography
IPF: idiopathic pulmonary fibrosis
MMP: matrix metalloproteinase
PaO_2_: partial pressure of oxygen in arterial blood
RT: room temperature
SDS: sodium dodecyl sulfate
TGF‐β: transforming growth factor beta
WHWT: West Highland white terrier

## 1 INTRODUCTION

Matrix metalloproteinases (MMPs) are a family of zinc‐containing endopeptidases. They are divided into subgroups based on substrate specificity and structure.^1^ Twenty‐three different MMPs have been identified in humans.^2^ These endopeptidases degrade both extracellular matrix (ECM) proteins, such as collagen and fibronectin, and non‐ECM proteins, such as cytokines and growth factors. Matrix metalloproteinases are secreted as inactive pro‐forms that require activation to form a functional active enzyme.^1^ In healthy tissue, MMP activity is low or absent, but it increases in pathological conditions, including lung diseases such as idiopathic pulmonary fibrosis (IPF) in humans^3^ and in physiological repair and remodeling processes.^1^

Canine IPF (CIPF) is a chronic, progressive, interstitial lung disease that mainly affects West Highland white terriers (WHWTs)^4^, ^5^ and occasionally other terrier breeds.^6^ This disease shares many similarities with IPF in humans, such as unknown etiology, clinical signs, and negative impact on survival.^6^, ^7^ High‐resolution computed tomography (HRCT) and histopathologic findings in CIPF are similar to those of both IPF and another interstitial lung disease in humans, non‐specific interstitial pneumonia.^8^, ^9^ Both CIPF and IPF are characterized by excessive deposition of ECM components in the lungs.^10^, ^11^ In humans, increased genetic expression, zymographic activity, immunoreactivity, or concentration of several MMPs are observed in lung tissue, bronchoalveolar lavage fluid (BALF), and blood of IPF patients.^3^ Of these MMPs, MMP‐7 is considered 1 of the best blood biomarkers for diagnosis and severity assessment of human IPF patients,^12^, ^13^ and it is a useful prognostic biomarker.^14^, ^15^ In addition to MMP‐7, gelatinolytic MMP‐2 and ‐9 also play a role in tissue remodeling in IPF of humans,^11^ and an increase of MMP‐9 in BALF reflects rapidly declining lung function.^16^

Matrix metalloproteinases, especially MMP‐9, are involved in several pulmonary diseases in dogs. In BALF, increased MMP‐9 but not MMP‐2 activity has been reported in dogs with eosinophilic bronchopneumopathy (EBP),^17^ recurrent bronchopneumonia, bronchiectasis,^18^ and in induced models of airway inflammation.^19^, ^20^ Increased pro‐MMP‐7 activity has been observed in the renal cortex of dogs with Alport syndrome^21^ and in the endometrium of dogs with cystic endometrial hyperplasia and pyometra.^22^ In addition, increased MMP‐7 expression has been observed in atrial tissue in induced atrial fibrillation in a Beagle dog model.^23^ To our knowledge, the only study of MMP‐7 in pulmonary diseases of dogs is a microarray study that identified downregulation of *MMP‐7* and also *MMP‐9* gene expression in lung tissue of dogs with CIPF.^24^

Our aim was to evaluate the diagnostic and prognostic value of MMP‐7 in serum and MMP‐2 and ‐9 in plasma and BALF in CIPF WHWTs and to compare findings with healthy dogs and dogs with other common respiratory diseases. We hypothesized that MMP activities would be increased in CIPF WHWTs and that, as in humans, especially blood MMP‐7 would be a potential diagnostic and prognostic marker for CIPF.

## 2 MATERIALS AND METHODS

### 2.1 Animals

In this cross‐sectional observational study, 44 CIPF WHWTs, 39 healthy WHWTs, 35 healthy dogs of other breeds, 24 dogs with chronic bronchitis (CB), 17 dogs with EBP, and 10 dogs with acute bacterial pneumonia (BP) were evaluated from January 2006 to June 2019. For CIPF and healthy WHWTs, follow‐up information (alive or dead, date and cause of death) was obtained either by contacting the owner or by retrieving the information from the patient information system at the study endpoint. The causes of death in WHWTs were divided into CIPF related (death or euthanasia because of acute respiratory signs or progressive worsening of CIPF signs) and non‐CIPF related. Survival times were calculated for both groups from study inclusion. Serum MMP‐7, BALF MMP‐2 and ‐9, and partial pressure of oxygen in arterial blood (PaO_2_) were evaluated as prognostic factors.

Diagnostic evaluation for dogs with respiratory disease and for healthy WHWTs consisted of thorough clinical examinations including all or most of the following: hematology, serum biochemistry, arterial blood gas analysis, fecal flotation and Baermann sedimentation test, thoracic radiographs, bronchoscopy with BALF sampling, and HRCT. In CIPF WHWTs, HRCT was performed in 22/44 dogs and postmortem lung histopathology was performed in 27/44 dogs. In 5/44 CIPF WHWTs, neither HRCT nor histopathology was performed, and the diagnosis was based on typical findings in clinical examination (crackles on lung auscultation and hypoxemia) and exclusion of other causes of exercise intolerance and cough. Some of the dogs in the CIPF group and group of healthy WHWTs had been included in previous clinical studies.^5^, ^25^, ^26^ All dogs in the different disease groups had clinical signs of lung disease. Dogs with CB had a history of cough with duration >2 months during a year. These dogs had a mild to moderate bronchointerstitial pattern on thoracic radiography and irregular bronchial epithelium and increased mucus formation noted in bronchoscopy. Other possible causes of cough in these dogs were excluded during diagnostic evaluation. The duration of signs in EBP dogs varied from 1 month to 2 years. Diagnosis was made by detecting sterile eosinophilic inflammation in BALF (eosinophils >20%) and by excluding other causes (eg, parasitic) for eosinophilia. All BP dogs had rapid onset of clinical signs (duration <2 days) and the diagnosis was based on radiographic findings (alveolar or interstitial consolidation), bacterial growth or intracellular bacteria in BALF (except in 1 dog detected by transtracheal wash), and response to antimicrobial treatment. In healthy dogs, no clinical findings suggestive of respiratory disease were observed in the 6 months before sampling. The health status of dogs of other breeds was verified by physical examination and routine blood test results. In healthy WHWTs, HRCT was performed in 31/39 dogs and postmortem lung histopathology was performed in 8/39 dogs. Exclusion criteria included doxycycline or systemic or inhaled glucocorticoid treatment in the 2 weeks before sampling and systemic diseases including infections, uncontrolled hyperadrenocorticism, and metastatic lung tumors.

Dogs with BP, CB, EBP, and healthy dogs of other breeds consisted of 46 different breeds and are listed in Table S1. Ages of dogs and sample storage times are shown in Table S2. All dogs were privately owned pet dogs except for 5 Beagles from the research colony of the University of Helsinki, Finland. These Beagles were cared for according to the principles outlined by the National Institutes of Health. Dogs were examined and samples were collected at the Veterinary Teaching Hospital of the University of Helsinki, Finland.

### 2.2 Sample collection

Serum and plasma samples were separated by centrifugation and stored at −80°C. Bronchoscopy was performed and BALF samples were processed as previously described.^27^ In brief, physiological saline (2 mL/kg/lobe divided in 2 aliquots) was used for collection of BALF samples from left and right caudal lung lobes during bronchoscopy. The supernatant was separated by centrifugation (100*g*, 10 minutes) and stored at −80°C.

The study protocol was approved by the Ethics Committee for Animal Experimentation at Helsinki University, Finland (statement numbers 5B/2008, 1/2014, and 4/2014) and by the Committee for Experimental Animals of Southern Finland (ESAVI/1005/04.10.03/2011, ESAVI/9116/04.10.07/2014, ESAVI/7383/04.10.07/2013, ESLH‐2008‐05403/Ym‐23, HY 132‐05). Owners of the pet dogs provided written consent to allow the use of the samples.

### 2.3 Matrix metalloproteinase activity analysis

All samples used were from the research sample bank of the Veterinary teaching hospital of Helsinki University. Based on sample availability, plasma, serum, BALF, or some combination of these was used. For MMP‐7 analysis, serum samples from 34 CIPF WHWTs, 32 healthy WHWTs, 35 healthy dogs of other breeds, 16 dogs with CB, 10 with EBP, and 10 with BP were included. For MMP‐2 and ‐9 analysis, plasma samples from 8 CIPF WHWTs and 9 healthy WHWTs and BALF samples from 17 CIPF WHWTs, 10 healthy WHWTs, 22 dogs with CB, and 16 dogs with EBP were used. The analyses performed in these groups also are listed in Table S3. The MMP activities were analyzed using optimal substrates in zymography (i.e., casein for MMP‐7 and gelatin for MMP‐2 and‐9).^28^

Casein zymography (modified from a previous study^21^) was performed on a 12% sodium dodecyl sulfate (SDS)‐polyacrylamide gel containing 1 mg/mL bovine β‐casein (Sigma Aldrich, St Louis, Missouri). Gels were first prerun at room temperature (RT) at a current of 40 mA until the bromophenol blue dye reached the bottom of the gel to remove excessive casein. Twenty microliter of serum (each sample loaded in duplicate) diluted 1:5 in 50 mM Tris‐HCl buffer (pH 7.5, containing 0.15 M NaCl, 0.01 M CaCl_2,_ and 0.05% Brij‐35), mixed with Laemmli buffer without reducing agent with a dilution of 1:2 was loaded to the gel. Each gel also was loaded with 10 ng of human recombinant MMP‐7 protein (Alpha Diagnostic International, San Antonio, Texas) and a molecular weight standard (Kaleidoscope, BioRad, Hercules, California). The gels were run in Tris/glycine SDS running buffer under RT conditions in an ice bath at a current of 20 mA for 10 minutes after the dye reached the end of the gel. The gels were washed for 1 hour in 2.5% Triton X‐100 to remove SDS and washed with 50 mM Tris‐HCl buffer (pH 7.5, containing 0.2 M NaCl, 0.005 M CaCl_2,_ and 0.02% Brij‐35) for 30 minutes and then incubated in the same buffer at 37°C for 21 hours. The gels then were rinsed, stained with Page Blue Protein staining solution (Thermo Scientific, Vilnius, Lithuania), and destained.

Gelatin zymography was performed on an 11% SDS‐polyacrylamide gel containing 0.7 mg/mL gelatin from porcine skin (Sigma Aldrich). Human recombinant MMP‐2 and ‐9 proteins (R&D Systems, Minneapolis, Minnesota) were used as positive controls. Gelatin zymography has been described previously.^17^, ^29^

**FIGURE 1:**
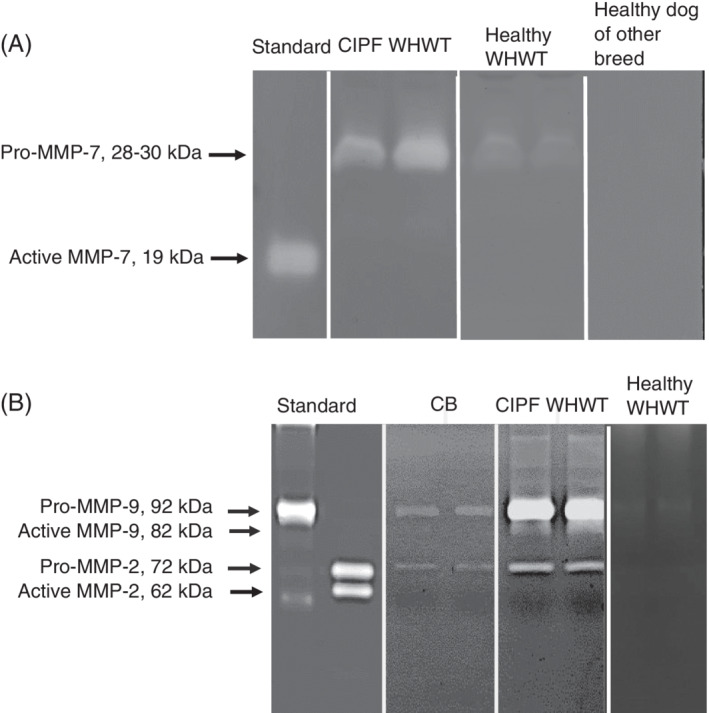
A, A representative image of a caseinolytic zymogram of serum samples. White bands of a West Highland white terrier (WHWT) with canine idiopathic pulmonary fibrosis (CIPF) and a healthy WHWT indicate pro‐matrix metalloproteinase (MMP)‐7 activity. No activity is detected in a sample from a healthy dog of other breed. B, A representative image of a gelatinolytic zymogram of bronchoalveolar lavage fluid. Both pro‐MMP‐2 and pro‐MMP‐9 activity are detected in the samples of a dog with chronic bronchitis (CB) and a WHWT with CIPF. Active MMP‐9 activity is present only in the sample of the CIPF WHWT

Caseinolytic and gelatinolytic activities were detected as clear bands against a dark background (Figure 1). For all MMPs, both pro‐form and active form were evaluated. The intensity of the band was quantified by the area mode of the Alpha Innotech program (Alpha Innotech, San Leandro, California). The area of each sample was normalized to the area of the band of human recombinant active MMP‐7 (serum), pro‐MMP‐9 (BALF), or pro‐MMP‐2 (plasma). The activity of each sample was reported as the mean of 2 parallel measurements.

To identify the caseinolytic band as pro‐MMP‐7, a Western blot assay was performed using MMP‐7 polyclonal antibody (Bioss Antibodies, dilution 1:300 and 1:1000) and horseradish peroxidase‐conjugated goat anti‐rabbit immunoglobulin (Dako, dilution 1:2000) for 2 serum samples. Western blots of MMP‐2 and ‐9 had been performed previously.^17^

### 2.4 Statistical analysis

Statistical analyses were performed using SAS System for Windows, version 9.4 (SAS Institute, Cary, North Carolina) and Graphpad Prism Version 8.0 for Windows (GraphPad Software, San Diego, California). *P* values <.05 were considered statistically significant. Normality was tested using the Shapiro‐Wilk test and normal *Q*‐*Q* plots. A Kruskal‐Wallis test was used to test group differences in MMP activities if >2 groups were compared and thereafter a Wilcoxon rank‐sum test with Bonferroni‐corrected *P* values was used for pairwise comparisons if group differences were detected. For plasma samples, a Wilcoxon rank‐sum test was used. The MMP‐2, ‐7, and ‐9 activities of CIPF WHWTs were compared with activities of other groups. In addition, MMP‐7 activities of healthy WHWTs were compared with activities of healthy dogs of other breeds.

Data from CIPF and healthy WHWTs were further analyzed by analysis of covariance (ANCOVA) using MMP activities as response variables. Age and storage time of samples were covariates and group (healthy WHWT, CIPF WHWT) as fixed effect. Square‐root transformation was applied on MMP results to satisfy the ANCOVA model normality assumption. Spearman correlation coefficient was used to examine the association between MMP activities and PaO_2_ in WHWTs.

Both all‐cause survival and CIPF‐specific survival of CIPF WHWTs against control WHWTs were compared using Kaplan‐Meier curves and estimated together using Cox proportional hazards analysis adjusted for the dog's age at the time of study inclusion. Dogs alive at study endpoint were censored from the all‐cause survival analyses. Furthermore, dogs that died of causes other than CIPF were censored from CIPF‐specific survival analysis. Cox regression was used to examine the effect of MMP activity and PaO_2_ on all‐cause and CIPF‐related deaths for the CIPF group. The model included the square‐root‐transformed MMP results, PaO_2_, and age as continuous predictors.

## 3 RESULTS

### 3.1 Matrix metalloproteinase activities

**FIGURE 2:**
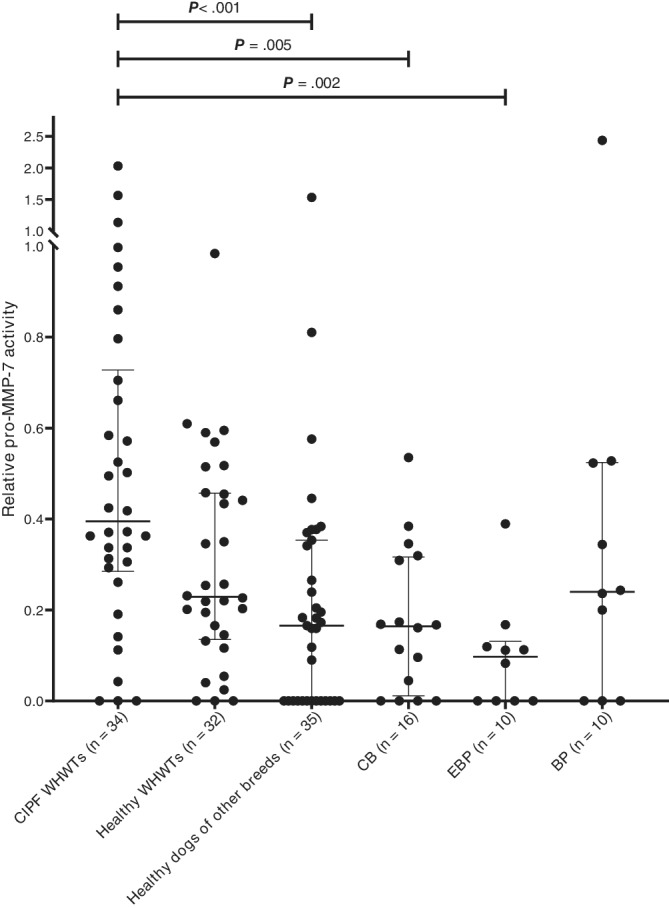
Scatter plot (median and interquartile range) of serum pro‐matrix metalloproteinase‐7 activities in West Highland white terriers with canine idiopathic pulmonary fibrosis compared with other groups. BP, bacterial pneumonia; CB, chronic bronchitis; CIPF, canine idiopathic pulmonary fibrosis, EBP, eosinophilic bronchopneumopathy; WHWT, West Highland white terrier

Serum pro‐MMP‐7 activities were significantly higher in CIPF WHWTs as compared with healthy dogs of other breeds, dogs with CB or dogs with EBP (Figure 2). No significant difference was detected in pro‐MMP‐7 activities between CIPF WHWTs and healthy WHWTs (*P* = .14) or between healthy WHWTs and healthy dogs of other breeds (*P* = .18). Active MMP‐7 was not detected in any of the samples. Caseinolytic proteinase was identified by Western blotting to be pro‐MMP‐7, with a molecular weight of 28 to 30 kDa.

**FIGURE 3:**
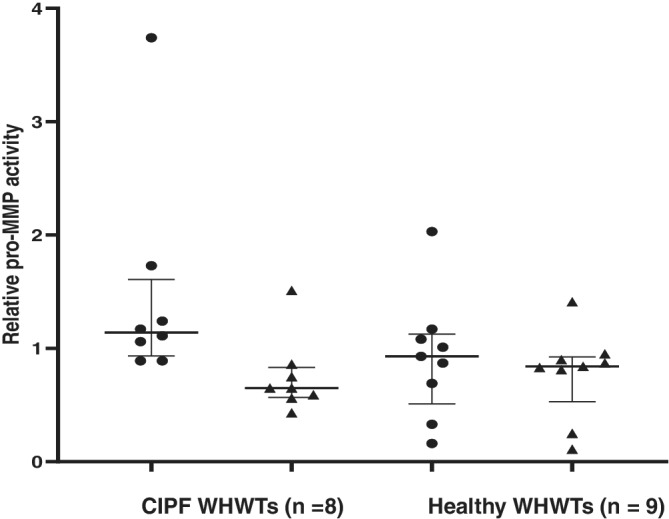
Scatter plot (median and interquartile range) of pro‐matrix metalloproteinase‐2 (triangles) and ‐9 (spots) activities in plasma of West Highland white terriers (WHWTs) with canine idiopathic pulmonary fibrosis (CIPF) and healthy WHWTs

No difference was detected between CIPF and healthy WHWTs in pro‐MMP‐2 (*P* = .37) or pro‐MMP‐9 (*P* = .12) activity in plasma (Figure 3). Active MMP‐9 was detected in plasma in 3/8 of CIPF WHWTs (median activity, 0; range, 0‐0.18) and in 6/9 of healthy WHWTs (median, 0.02; range, 0‐0.08) but no statistically significant difference was detected (*P* = .76). Active MMP‐2 was not detected in any of the plasma samples.

**FIGURE 4:**
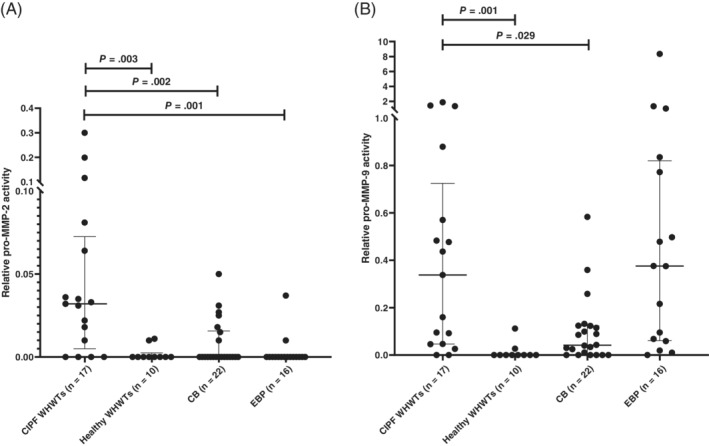
A, Scatter plot (median and interquartile range) of bronchoalveolar lavage fluid pro‐matrix metalloproteinase (MMP)‐2 activities in West Highland white terriers with canine idiopathic pulmonary fibrosis compared with other groups. B, Scatter plot (median and interquartile range) of bronchoalveolar lavage fluid pro‐MMP‐9 activities in West Highland white terriers with CIPF compared with other groups. CB, chronic bronchitis; CIPF, canine idiopathic pulmonary fibrosis; EBP, eosinophilic bronchopneumopathy; WHWT, West Highland white terrier

In BALF, pro‐MMP‐2 activities were significantly increased in CIPF WHWTs compared with healthy WHWTs, dogs with CB or those with EBP (Figure 4A). Activities of pro‐MMP‐9 in BALF were significantly higher in CIPF WHWTs than in healthy WHWTs and dogs with CB (Figure 4 B), but no difference was detected between CIPF WHWTs and EBP dogs (*P* > 1). Active MMP‐9 was detected in BALF in 5/17 of dogs with CIPF (median activity, 0; range, 0‐0.84) and in 5/16 of dogs with EBP (median, 0; range, 0‐1.12), but not in the other groups. Active MMP‐2 was detected only in 1/17 CIPF WHWTs.

In the ANCOVA model of WHWTs, no association (*P* > .05) was detected between serum pro‐MMP‐7 and age or storage time, nor between BALF pro‐MMP‐2 and ‐9 and age or storage time. Instead, plasma pro‐MMP‐2 and ‐9 had significant negative association (*P* < .05) with storage time (estimate, −0.24 per 500 days of storage; 95% confidence interval [CI], −0.45 to −0.03; estimate, −0.32; 95% CI, −0.61 to −0.04) but not with age (*P* > .05).

In CIPF WHWTs, median PaO_2_ was 59 mmHg (range, 39‐87 mmHg; n = 35) and in healthy WHWTs PaO_2_ was 95 mmHg (range, 86‐113 mmHg; n = 36). In all WHWTs, a mild negative correlation was found between serum pro‐MMP‐7 activity and PaO_2_ (*r*_S_ = −0.28; *P* = .03) and a moderate negative correlation was found between BALF pro‐MMP‐9 and PaO_2_ (*r*_S_ = −0.67; *P* = .0003) and between BALF pro‐MMP‐2 and PaO2 (*r*_S_ = −0.62; *P* = .001). When only CIPF WHWTs were evaluated, no correlations were detected between serum pro‐MMP‐7 and PaO_2_ (*r*_S_ = 0.13; *P* = .5), BALF pro‐MMP‐9 and PaO_2_ (*r*_S_ = −0.33; *P* = .25), or BALF pro‐MMP‐2 and PaO_2_ (*r*_S_ = −0.44; *P* = .12)_._

### 3.2 Survival and prognostic factor analysis

**FIGURE 5:**
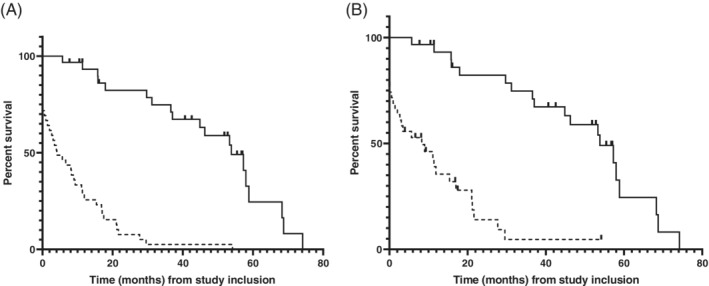
A, Kaplan–Meier survival curves for all‐cause survival of West Highland white terriers (WHWTs) with canine idiopathic pulmonary fibrosis (CIPF, n = 39, solid line), and control WHWTs (n = 31, dashed line) from time of study inclusion. Censored animals (control WHWTs alive at study endpoint) are presented as dashed lines. B, Kaplan‐Meier survival curves for CIPF‐specific survival of WHWTs with CIPF (n = 39, dashed line), and control WHWTs (n = 31, solid line) from time of study inclusion. Censored animals (control WHWTs alive at study endpoint and CIPF WHWTs died because non‐CIPF‐related cause) are presented as zaps

Information about the status of the dog (dead or alive) was available for 39/44 of CIPF WHWTs and for 31/39 of healthy WHWTs. Median follow‐up period was 16 months (range, 0‐74 months). During the follow‐up period, all of the CIPF WHWTs died or were euthanized (30/39 because of CIPF, 7/39 for another cause and 2/39 for unknown reason). In the control WHWT group, 19/31 died or were euthanized for non‐CIPF‐related causes and those dogs (12/31), that were alive at study endpoint, had no signs of pulmonary diseases. Median (adjusted for censored data) all‐cause survival in WHWTs with CIPF was 4 months (95% CI, 1‐9 months) and median CIPF‐specific survival was 8 months (95% CI, 1‐15 months) from study inclusion. Median survival of control WHWTs that died was 54 months (95% CI, 37‐59 months) from study inclusion. Based on the Cox regression model for all‐cause survival, the hazard ratio (HR) for risk of death in WHWTs with CIPF from study inclusion compared to control WHWTs was 5.7 (95% CI, 2.7‐12.1; *P* < .0001). When CIPF‐specific deaths of CIPF WHWTs were compared to control WHWTs, the HR for risk of death was 4.2 (95% CI, 1.9‐9.4; *P* = .0004). Kaplan‐Meier curves for survival of WHWTs with CIPF and control WHWTs from study inclusion are presented in Figure 5A,B.

No statistically significant prognostic factors associated with survival in CIPF WHWTs were identified (Table 1). When only CIPF WHWTs with PaO_2_ ≤ 60 mmHg were evaluated, serum pro‐MMP‐7 activity was significantly associated with increased risk of death in all‐cause related deaths (n = 14; HR, 1.31; 95% CI, 1.01‐1.69; *P* = .04) and in CIPF‐related deaths (n = 10; HR, 1.46; 95% CI, 1.03‐2.06; *P* = .03).

**TABLE 1:** Effect of prognostic factors in Cox proportional hazard analysis on CIPF‐related and all‐cause‐related deaths in West Highland white terriers

Variables (unit change)	All‐cause related death, HR (95% CI)	N	*P*	CIPF‐related death, HR (95% CI)	N	*P*
Serum pro‐MMP‐7 (.1)	1.08 (0.90‐1.29)	23	.48	1.09 (0.89‐1.32)	19	.42
BALF pro‐MMP‐2 (.1)	0.98 (0.69‐1.41)	13	.93	0.98 (0.67‐1.43)	10	.90
BALF pro‐MMP‐9 (.1)	0.98 (0.84‐1.15)	13	.80	0.95 (0.80‐1.13)	10	.55
PaO_2_ (10)	1.11 (0.74‐1.66)	31	.62	1.03 (0.65‐1.64)	23	.91

*Note:* Results are from separate statistical models. Models included square‐root‐transformed MMP activities, PaO_2_, and age as continuous predictors. PaO_2_ results are from the model including PaO_2_ and age only.

Abbreviations: BALF, bronchoalveolar lavage fluid; CI, confidence interval; CIPF, canine idiopathic pulmonary fibrosis; HR, hazard ratio; MMP, matrix metalloproteinase; PaO_2_, partial pressure of oxygen in arterial blood.

## 4 DISCUSSION

We compared the activities of MMP‐7 in blood and MMP‐2 and ‐9 in BALF of CIPF dogs, dogs with other lung diseases, and healthy dogs. In addition, we evaluated plasma MMP‐2 and ‐9 activities in CIPF and healthy WHWTs. Our findings suggest that especially serum MMP‐7 but also BALF MMP‐2 and ‐9 may be potential diagnostic but not prognostic markers for CIPF.

Matrix metalloproteinases are secreted in latent pro‐forms. Activation requires removal of a cysteine residue (ie, cysteine switch), which creates fully active MMP with molecular weight approximately 10 kDa lower than for the pro‐form. Synthesis and activity of MMPs are regulated at several stages starting from gene transcription and ending with active MMP degradation.^28^ We evaluated MMP activities by using zymography, which allows the detection of both pro‐form and active form. Higher activity of both pro‐MMP and active MMP in comparison with control or other disease groups reflects changes in proteolytic capacity which are absent or at low concentration in healthy tissue.^28^

Serum pro‐MMP‐7 activity was significantly higher in CIPF WHWTs than in dogs with chronic lung diseases (ie, CB, EBP) or healthy dogs of other breeds but not when compared with dogs with acute BP. Although our findings concur with several studies in humans, no previous reports of blood MMP‐7 in any lung diseases of dogs exist to our knowledge. Studies of humans have identified increased total MMP‐7 (including both pro‐form and active form) concentrations in the blood of IPF patients when compared with healthy controls, patients with other interstitial lung diseases, or those with chronic obstructive pulmonary disease.^12^, ^14^, ^15^, ^30^ In humans, MMP‐7 is considered a profibrotic mediator^3^ and a potential blood prognostic and diagnostic biomarker for IPF.^12^, ^13^, ^14^, ^15^ The profibrotic nature of MMP‐7 also is supported by findings in animal models and in vitro. Mice that are MMP‐7 deficient are protected from bleomycin‐induced lung fibrosis.^31^ Matrix metalloproteinase‐7 degrades several ECM components and regulates transforming growth factor beta (TGF‐β) bioactivity,^32^ which is a key mediator in CIPF of WHWTs.^33^, ^34^ Inconsistent with our hypothesis, we did not observe an association between PaO_2_ (ie, severity of CIPF) and pro‐MMP‐7 activity in correlation analysis in CIPF WHWTs. This observation might indicate that serum pro‐MMP‐7 activity has limited utility as a marker of disease severity. This finding is in contrast to IPF in humans, where increasing total MMP‐7 concentrations are associated with decreasing lung function.^12^ Our BP dogs also had high pro‐MMP‐7 activities, which may be related to the diverse functions of MMP‐7. One function is regulating neutrophil recruitment at the site of acute lung injury.^35^ Serum pro‐MMP‐7 activity was significantly higher in CIPF WHWTs than in healthy dogs of other breeds, but not when compared with healthy WHWTs. One explanation might be that even though healthy WHWTs were thoroughly examined and thought to have healthy lungs, they may have had subclinical, early phase fibrosis with undetectable findings on HRCT that have not yet caused hypoxemia. In humans, serum MMP‐7 is considered potentially valuable for detection of subclinical interstitial lung disease.^30^, ^36^ In addition, the possible effect of other organ fibrosis on pro‐MMP‐7 activity cannot be excluded. In humans, increased blood total MMP‐7 concentrations are associated with severe fibrosis of other organs, such as kidney^37^ and liver.^38^ In our study, necropsy was performed in 27/44 CIPF WHWTs, but only in a few dogs in other groups. Therefore, we were not able to evaluate whether fibrosis in other organs might have affected serum pro‐MMP‐7 activity. The lack of difference in serum pro‐MMP‐7 activities between CIPF and healthy WHWTs also might be explained as a breed specific finding. In addition, our study may have been underpowered to identify a significant difference. A previous study observed downregulation of *MMP‐7* gene expression by microarray in lung samples of dogs with CIPF compared to healthy controls.^24^ However, the analysis method and sample type (lung tissue) were different and it is known that posttranscriptional, translational, and degradation regulation affect protein abundance.^39^ In addition, the previous used pooled samples, reported of low probe signal intensities and did not confirm results by qRT‐PCR.^24^

In BALF, pro‐MMP‐2 activity was significantly higher in CIPF WHWTs compared with other groups (healthy WHWTs, CB, and EBP groups). In a previous study, pro‐MMP‐2 activity was not significantly higher in dogs with EBP as compared to healthy controls.^17^ Increased pro‐MMP‐2 activity has been detected in BALF of rats with bleomycin‐induced fibrosis.^40^ In addition, increased total MMP‐2 concentrations have been observed in BALF of human patients with IPF compared with healthy controls.^16^ The MMP‐2 activity may be linked with basement membrane degradation^41^ and angiogenesis,^42^ which in turn may promote alveolar fibrosis.^16^ In humans with IPF, MMP‐2 is mainly expressed in reactive airway epithelial cells and myofibroblasts, but also in inflammatory cells such as macrophages.^43^

The activity of pro‐MMP‐9 in BALF was significantly increased in CIPF WHWTs compared with healthy WHWTs and dogs with CB. Total MMP‐9 concentrations also increased in BALF in humans with IPF compared to healthy controls.^16^ We did not detect a significant difference between CIPF WHWTs and dogs with EBP. Increased pro‐MMP‐9 and active MMP‐9 activity previously has been detected in dogs with EBP compared with healthy controls.^17^ Similarly, increased MMP‐9 activities have been detected in dogs with recurrent bronchopneumonia and bronchiectasis^18^ and in canine models of airway inflammation.^19^, ^20^ As was the case with MMP‐7, no correlations were found between pro‐MMP‐2 or pro‐MMP‐9 activity in BALF and PaO_2_ when only the CIPF group was evaluated, indicating that these MMPs also may have limited utility as severity markers. In humans, high BALF total MMP‐9 concentrations were found in IPF patients with rapid disease progression in a study with small groups of both diseased and control subjects.^16^ Bleomycin‐induced pulmonary fibrosis in a rat model showed that MMP‐2 and ‐9 may have important roles in the early phase of the disease. In the later phase, these MMPs also may have a role in the repair process.^40^ Active MMP‐9 was detected only in 5 CIPF WHWTs and in 5 dogs with EPB, and active MMP‐2 was only detected in 1 CIPF WHWT. An experimental study of rats with lung fibrosis found that active forms of MMPs more often are present in lung tissue, where protein degradation occurs, than in BALF.^40^ In addition, active MMP in BALF might be too low to be detected by zymography. Matrix metalloproteinase‐9 promotes abnormal epithelial repair in humans with IPF because it is mostly expressed in alveolar macrophages, metaplastic airway epithelial cells, and neutrophils^44^ and because it activates latent TGF‐β.^45^ A microarray study observed downregulation of the *MMP‐9* gene in the lungs of dogs with CIPF.^24^ As described earlier, the method and matrix differed from those used in our study.

In contrast to the BALF results, we did not observe any significant difference in plasma pro‐MMP‐2 and ‐9 activities between CIPF and healthy WHWTs. Active MMP‐9 was detected both in CIPF and healthy WHWTs, but no significant difference was observed between groups with limited numbers of dogs. However, blood and BALF MMP concentrations are not always comparable with each other.^46^ In humans, increased blood total MMP‐2 and ‐9 concentrations have been detected in IPF patients compared to healthy controls.^13^ Furthermore, increased pro‐MMP‐9, active MMP‐9, and active MMP‐2 activity has been observed in patients with severe IPF compared to healthy controls, but no significant difference was observed when patients with moderate IPF were compared with healthy controls.^47^ An ANCOVA model of WHWTs showed that sample storage time may decrease pro‐MMP‐2 and ‐9 activities in plasma. In humans, plasma MMP‐9 concentrations have been shown to be stable after 9 years of storage at −80°C.^48^ Conflicting results have been published,^49^ however, which was our rationale for including storage time in the ANCOVA model of WHWTs.

We studied survival of CIPF WHWTs in comparison to healthy WHWTs and evaluated whether MMP activities and PaO_2_ could serve as prognostic factors for CIPF. Not surprisingly, median survival time differed between CIPF and healthy WHWTs. For all‐cause survival, the risk of death from the time of study inclusion was 5.7 times higher in CIPF WHWTs compared to control WHWTs when age was adjusted in the statistical model. Similar results have been reported previously with a 4.4‐fold higher risk of death in CIPF WHWTs compared to control WHWTs.^25^ We did not observe that MMP activities or PaO_2_ were prognostic for CIPF‐related death. Another study had similar findings regarding PaO_2_.^25^ However, in a subgroup of CIPF WHWTs with advanced disease (PaO_2_ ≤ 60 mmHg), serum pro‐MMP‐7 activity was associated with increased risk of death even though the number of dogs was small. In human patients, several lung function parameters^50^ and total MMP‐7 in blood^15^, ^51^ are potential prognostic indicators for IPF.

In addition to other organ fibrosis, several other factors can have an effect on blood MMP concentrations such as malignant tumors and obesity. Obesity can increase blood MMP‐2 and ‐9 concentrations in humans.^52^ None of our WHWTs were obese, based on body condition scores. In humans, several malignant tumors, such as pancreatic adenocarcinoma, metastatic renal cell tumor and colorectal cancer, can increase blood MMP‐2, ‐7, or ‐9 concentrations.^53^ Dogs in our study had no clinical findings indicating the presence of malignant tumors.

Our study had some limitations. Some groups were small, especially with respect to plasma samples. Samples were not available for complete analysis both from BALF and blood in all groups. Because of the preliminary nature of our study investigating MMP activities for the first time in CIPF, power analysis was not performed. Healthy WHWTs may have been in the early stages of CIPF, which was not detected in HRCT, but their inclusion was of interest as a predisposed breed. Additionally, human recombinant proteins and antibodies used in Western blotting have not been validated for dogs.

In conclusion, our results suggest that especially circulating MMP‐7 but also BALF MMP‐2 and ‐9 may be potential diagnostic markers for CIPF. However, these MMPs do not seem to be potential prognostic markers for CIPF.

## CONFLICT OF INTEREST

Authors declare no conflict of interest.

## OFF‐LABEL ANTIMICROBIAL DECLARATION

Authors declare no off‐label use of antimicrobials.

## INSTITUTIONAL ANIMAL CARE AND USE COMMITTEE (IACUC) OR OTHER APPROVAL DECLARATION

Approved by the Ethics Committee for Animal Experimentation at Helsinki University, Finland (statement numbers 5B/2008, 1/2014, and 4/2014) and by the Committee for Experimental Animals of Southern Finland (ESAVI/1005/04.10.03/2011, ESAVI/9116/04.10.07/2014, ESAVI/7383/04.10.07/2013, ESLH‐2008‐05403/Ym‐23, HY 132‐05).

## HUMAN ETHICS APPROVAL DECLARATION

Authors declare human ethics approval was not needed for this study.

## Supporting information


**Table S1** Dog breeds in groups of chronic bronchitis, eosinophilic bronchopneumopathy, bacterial pneumonia, and healthy dogs of other breeds

**Table S2** Ages of dogs and storage times of blood and bronchoalveolar lavage samples in different dog groups

**Table S3** Dog groups used in different matrix metalloproteinase (MMP) activity analyses
