# Supplementary material for: Matrix metalloproteinase‐2, ‐7, and ‐9 activities in dogs with idiopathic pulmonary fibrosis compared to healthy dogs and dogs with other respiratory diseases
Source: J Vet Intern Med. 2020 Dec 4;35(1):462–71. doi: 10.1111/jvim.15970 (PMC7848316; doi:10.1111/jvim.15970)
Supplement: Supplementary file 1 — Table S1 Dog breeds in groups of chronic bronchitis, eosinophilic bronchopneumopathy, bacterial pneumonia, and healthy dogs of other breeds [file JVIM-35-462-s001.pdf]

Supporting Information 1.

Dog breeds in groups of chronic bronchitis, eosinophilic bronchopneumopathy, bacterial pneumonia, and healthy dogs of other breeds.

	**CB**	**EBP**	**BP**	**Healthy dogs of other breeds**
**Serum**	Shetland sheepdog (n=5)<br><br>Chinese crested dog (n=2)<br><br>Bichon frise (n=2)<br><br>Smooth fox terrier<br><br>Cavalier King Charles spaniel<br><br>Hungarian greyhound<br><br>Cairnterrier<br><br>Welsh corgi<br><br>Mixed breed<br><br>Jack Russell terrier	Mixed breed (n=2)<br><br>Australian shepherd<br><br>Tibetan mastiff<br><br>Finnish lapphund<br><br>Borderterrier<br><br>Afghan hound<br><br>Rottweiler<br><br>Labrador retriever<br><br>Affenpinscher	Irish wolfhound (n=2)<br><br>Bullmastiff<br><br>Russian greyhound<br><br>Labrador retriever<br><br>German shepherd<br><br>Dobermann<br><br>Scottish deerhound<br><br>Pharaoh hound	Mixed breed (n=7)<br><br>Beagle (n=5)<br><br>German shepherd (n=3)<br><br>Whippet (n=3)<br><br>Russian greyhound<br><br>Rottweiler<br><br>Australian shepherd<br><br>Spanish greyhound<br><br>Bichon havanais<br><br>Finnish lapphund<br><br>Lapponian herder<br><br>Miniature pinscher<br><br>Bordercollie<br><br>Miniature poodle<br><br>Labrador retriever<br><br>Flat coated retriever<br><br>Bolognese<br><br>Australian cattle dog<br><br>Giant schnauzer<br><br>Golden retriever

**BALF**	Shetland sheepdog (n=7)<br><br>Labrador retriever<br><br>English springerspaniel<br><br>Wheaten terrier<br><br>Griffon bruxellois<br><br>Cairnterrier<br><br>Staffordshire terrier<br><br>Golden retriever<br><br>Smooth fox terrier<br><br>Cavalier King Charles spaniel<br><br>Mixed breed<br><br>Welsh corgi<br><br>Bichon frise<br><br>Jack Russell terrier<br><br>Hungarian greyhound	Labrador retriever (n=3)<br><br>Mixed breed (n=2)<br><br>Lapponian herder<br><br>Nova Scotia duck tolling retriever<br><br>Small Münsterländer<br><br>Siberian husky<br><br>Borderterrier<br><br>Affenpinscher<br><br>Tibetan mastiff<br><br>Australian shepherd<br><br>Afghan hound<br><br>Rottweiler		

BALF, bronchoalveolar lavage fluid; BP, bacterial pneumonia; CB, chronic bronchitis; EBP, eosinophilic bronchopneumopathy
