# Supplementary material for: Matrix metalloproteinase‐2, ‐7, and ‐9 activities in dogs with idiopathic pulmonary fibrosis compared to healthy dogs and dogs with other respiratory diseases
Source: J Vet Intern Med. 2020 Dec 4;35(1):462–71. doi: 10.1111/jvim.15970 (PMC7848316; doi:10.1111/jvim.15970)
Supplement: Supplementary file 2 — Table S2 Ages of dogs and storage times of blood and bronchoalveolar lavage samples in different dog groups [file JVIM-35-462-s002.docx]

**Supporting Information 2.**

Ages of dogs and storage times of blood and bronchoalveolar lavage samples in different dog groups.

| **Groups** | **Age** (years), median (range) | **Storage time** (days), median (range) |
| --- | --- | --- |
| CIPF WHWTs (n=44) | 12.0 (8.0-16.3) | 1240 (20-3993) |
| Healthy WHWTs (n=39) | 9.8 (7.3-14.0) | 1182 (17-3374) |
| Healthy dogs of other breeds (n=35) | 10.4 (4.5-15.1) | 13 (1-2591) |
| CB (n=24) | 9.9 (3.7-13.9) | 1567 (19-4289) |
| EBP (n=17) | 3.1 (0.7-8.5) | 706 (159-2757) |
| BP (n=10) | 5.7 (0.7-11.8) | 2350 (2044-2665) |

BP, bacterial pneumonia; CB, chronic bronchitis; CIPF, canine idiopathic pulmonary fibrosis; EBP, eosinophilic bronchopneumopathy; WHWT, West Highland white terrier.
