# Supplementary material for: Matrix metalloproteinase‐2, ‐7, and ‐9 activities in dogs with idiopathic pulmonary fibrosis compared to healthy dogs and dogs with other respiratory diseases
Source: J Vet Intern Med. 2020 Dec 4;35(1):462–71. doi: 10.1111/jvim.15970 (PMC7848316; doi:10.1111/jvim.15970)
Supplement: Supplementary file 3 — Table S3 Dog groups used in different matrix metalloproteinase (MMP) activity analyses [file JVIM-35-462-s003.pdf]

Supporting Information 3.

Dog groups used in different matrix metalloproteinase (MMP) activity analyses.

**Groups**	**Serum MMP-7 (n)**	**BALF MMP-2 and -9 (n)**	**Plasma MMP-2 and -9 (n)**
CIPF WHWTs	34	17	8
Dogs with CB	16	22	
Dogs with EBP	10	16	
Dogs with BP	10		
Healthy WHWTs	32	10	9
Healthy dogs of other breeds	35		

BALF, bronchoalveolar lavage fluid; BP, bacterial pneumonia; CB, chronic bronchitis; CIPF, canine idiopathic pulmonary fibrosis; EBP, eosinophilic bronchopneumopathy; WHWT, West Highland white terrier
